# Three New Sesquiterpene Glycosides from the Rhizomes of *Trillium tschonoskii*

**DOI:** 10.3390/molecules22081283

**Published:** 2017-08-02

**Authors:** Jie Yang, Yin-Jun Yang, Xin-Guang Sun, Jie Zhang, Yang Zhao, Bei Wang, Qian-Zhi Ding, Bao-Lin Guo, Bai-Ping Ma

**Affiliations:** 1 School of Traditional Chinese Medicine, Guangdong Pharmaceutical University, Guangzhou 510006, China; yangjie0528@163.com (J.Y.); wbshell125@126.com (B.W.); 2 Beijing Institute of Radiation Medicine, No. 27, Taiping Road, Beijing 100850, China; iyangyinjun@163.com (Y.-J.Y.); sxgzhwu07@163.com (X.-G.S.); zhangjie061003@163.com (J.Z.); mmyzhao@163.com (Y.Z.); dqz1990@163.com (Q.-Z.D.); 3 Institute of Medicinal Plant Development, Chinese Academy of Medical Sciences, Beijing 100193, China; guobaolin010@163.com

**Keywords:** *Trillium tschonoskii*, sesquiterpene glycosides, separation

## Abstract

Three new sesquiterpene glycosides, possessing a rare aglycone with a sulfonyl between C-1 and C-15 positions, named 3-(3′*E*-7′*R*,8′-dihydroxy-4′,8′-dimethyl-3′-nonenyl)-2,5-dihydro-1,1-dioxo-thiophen 7′-*O*-β-d-glucopyranosyl-(1→4)-*O*-β-d-glucopyranosyl-(1→4)-*O*-β-d-glucopyranoside (**1**), 3-(3′*E*-7′*R*,8′-dihydroxy-4′,8′-dimethyl-3′-nonenyl)-2,5-dihydro-1,1-dioxo-thiophen 7′-*O*-β-d-glucopyranosyl-(1→4)-*O*-β-d-glucopyranoside (**2**), and 3-(3′*E*-7′*R*,8′-dihydroxy-4′,8′-dimethyl-3′-nonenyl)-2,5-dihydro-1,1-dioxo-thiophen 7′-*O*-β-d-glucopyranosyl-6′-*O*-acetyl-(1→4)-*O*-β-d-glucopyranosyl-(1→4)-*O*-β-d-glucopyranoside (**3**), respectively, were isolated from the rhizomes of *Trillium tschonoskii*. Their structures were established on the basis of spectroscopic data, including HR-ESI-MS, IR, 1D and 2D NMR. The cytotoxic properties of the three compounds were investigated using human hepatic L02 cells.

## 1. Introduction

*Trillium tschonoskii* Maxim is perennial herb, mainly distributed in Hubei, Shanxi and Anhui provinces of China at an altitude of 1600–3200 m [1]. The rhizomes of *T. tschonoskii* named Yan Ling Cao, have been used as a traditional Chinese medicine (TMC) for the treatment of headache, traumatic injury, and neurasthenia [2]. Recent pharmacological studies have shown that Yan Ling Cao possesses anti-tumor, anti-inflammatory, analgesic and blood coagulation activities [3,4,5,6,7]. Previous phytochemical studies on the *Trillium* reveals that steroidal saponins constitute the main chemical components [6,8,9,10,11,12]. Besides, it also contains a few phenylpropanoid glycosides [13] and sesquiterpenoid glycosides [3,13,14]. In this study, a chemical investigation on *T. tschonoskii* led to the isolation of three new sesquiterpene glycosides that have a rare aglycone with a sulfonyl between C-1 and C-15 positions. Their structures were identified using the spectroscopic techniques of HR-ESIMS, IR, and NMR. The cytotoxic activity of the three compounds were evaluated against L02 cells.

## 2. Results and Discussion

**Figure 1:**
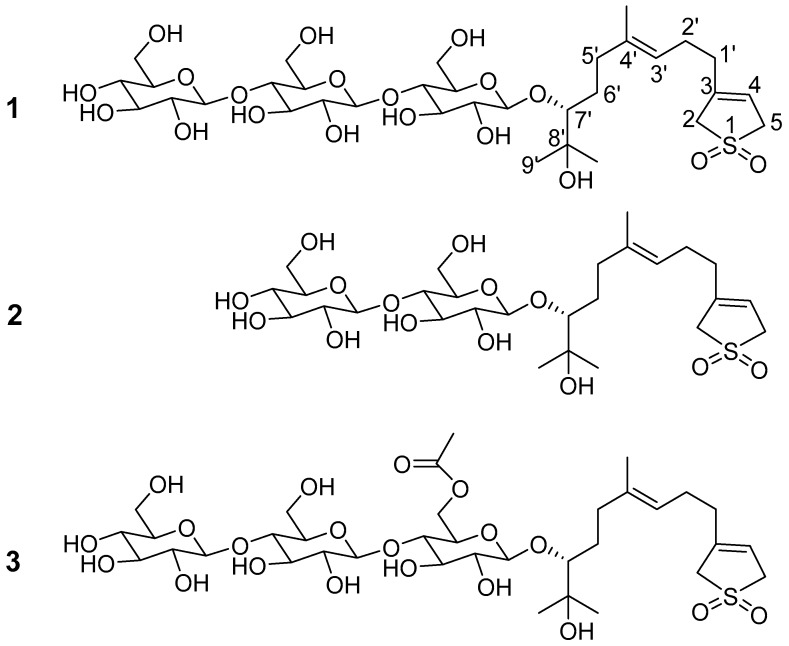
The chemical structures of compounds **1**–**3**.

The rhizomes of *T. tschonoskii* were extracted using 50% aq. EtOH. The extract was subjected to macroporous resin SP825 column chromatography to afford five fractions (Fr. A−Fr. E). Fraction C was subsequently separated on silica-gel, MCI, ODS, preparative and semi-preparative HPLC to provide three new sesquiterpene glycosides (Figure 1), named 3-(3′*E*-7′*R*,8′-dihydroxy-4′,8′-dimethyl-3′-nonenyl)-2,5-dihydro-1,1-dioxo-thiophen 7′-*O*-β-d-glucopyranosyl-(1→4)-*O*-β-d-glucopyranosyl-(1→4)-*O*-β-d-glucopyranoside (**1**), 3-(3′*E*-7′*R*,8′-dihydroxy-4′,8′-dimethyl-3′-nonenyl)-2,5-dihydro-1,1-dioxo-thiophen 7′-*O*-β-d-glucopyranosyl-(1→4)-*O*-β-d-glucopyranoside (**2**), 3-(3′*E*-7′*R*,8′-dihydroxy-4′,8′-dimethyl-3′-nonenyl)-2,5-dihydro-1,1-dioxo-thiophen 7′-*O*-β-d-glucopyranosyl-6′-*O*-acetyl-(1→4)-*O*-β-d-glucopyranosyl-(1→4)-*O*-β-d-glucopyranoside (**3**), which were identified by NMR, IR and HR-ESI-MS techniques.

**Figure 2:**
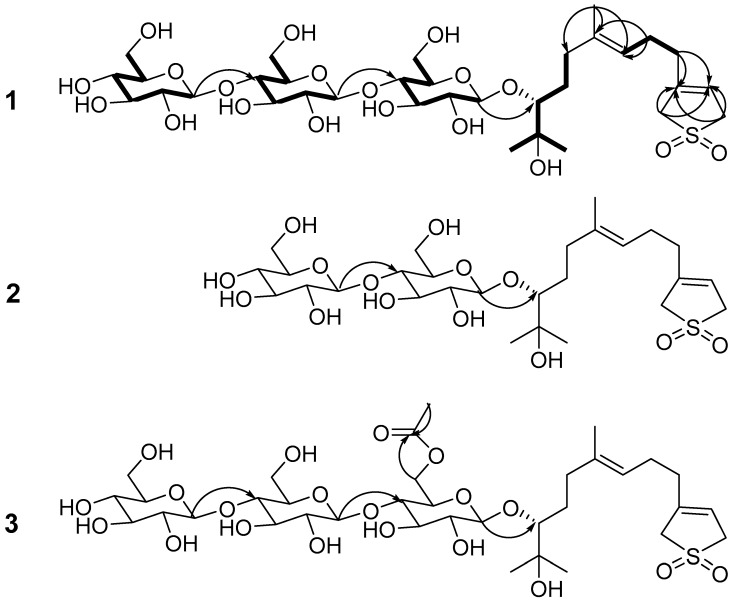
Key HMBC (arrows) and ^1^H−^1^H COSY (thick lines) correlations of compounds **1**–**3**.

Compound **1** was obtained as a white amorphous powder and the molecular formula C_33_H_56_O_19_S was indicated by HR-ESIMS at *m*/*z* 787.3065 [M − H]^−^ (calcd. for C_33_H_55_O_19_S 787.3058). The ^1^H-NMR (600 MHz) spectrum of **1** (Table 1) showed three tertiary methyl group signals (δ_H_ 1.37, 1.32, 1.61), two olefinic protons (δ_H_ 5.58, 5.34 (br t, *J* = 7.2 Hz)), as well as signals for three anomeric protons at (δ_H_ 4.89 (d, *J* = 7.9 Hz), 5.12 (d, *J* = 7.8 Hz), and 5.15 (d, *J* = 7.8 Hz)). The ^13^C combined with HSQC NMR spectra of **1** indicated a structure with a total of 33 C-atom signals. Fifteen of them were attributed to the aglycone carbons including four olefinic carbons (δ_C_ 117.8, 138.7, 123.6, 136.8), one oxygenated methine carbon (δ_C_ 90.0), one oxygenated quaternary carbon (δ_C_ 71.9), two sulfonated methylene carbons (δ_C_ 57.4, 58.0), four sp3 methylene carbons (δ_C_ 25.6, 33.1, 36.2, 30.8), and three tertiary methyl carbons (δ_C_ 16.1, 25.3, 26.8), while the remaining carbon signals were characteristic to three glucosyl moieties. By comparing the NMR data of **1** with 3-(3′*E*-7′,8,-dihydroxy-4′,8′-dimethyl-3′-nonenyl)-2,5-dihydro-1,1-dioxo-thiophen [15], the structure of **1** was similar to 3-(3′*E*-7′,8,-dihydroxy-4′,8′-dimethyl-3′-nonenyl)-2,5-dihydro-1,1-dioxo-thiophen except for one sugar moiety at C-10 of 1, which was further supported by ^1^H−^1^H COSY correlation (Figure 2) of H-1/H-2, H-5/H-4 and H-6, H-9/H-8 and H-10, and HMBC correlation of H-2/C-3, C-4 and C-15, H-6/C-8 and C-14, H-10/C-11 and C-12, H-12/C-10, C-11 and C-13. Comparison the NMR data of **1** with (2,3-*S*-*trans*,10*R*,6*E*)-7,11-dimethyl-3-methylene-1,6-dodecadien-10,11-diol 10-*O*-β-d-glucopyranosyl-(1→4)-*O*-β-d-glucopyranosyl-(1→4)-*O*-β-d-glucopyranoside [14] suggested that they shared the same sugar chain. The sugar moiety was further assigned by HSQC, HMBC and ^1^H–^1^H COSY experiments. Furthermore, the HMBC correlations between H-1-Glc′ (δ 4.89) and C-10 (δ 90.0), H-1-Glc″ (δ 5.12) and C-4-Glc′″ (δ 80.9), H-1-Glc′ (δ 5.15) and C-4-Glc″ (δ 80.9) (Figure 2) verified that the linkage of the sugar unit and its location at C-10 of **1**. The absolute configuration at C-10 of **1** was confirmed as *R* by the values of glycosylation shift of α-, β-(pro-*S* side), and β-(pro-*R* side) carbons of secondary alcohols to which glucosyl moieties were attached [16]. Furthermore, the ^13^C chemical shifts at C-8 and C-12 of **1** were quite similar to those of (2,3-*S*-trans,10*R*,6*E*)-7,11-dimethyl-3-methylene-1,6-dodecadien-10,11-diol 10-*O*-β-d-glucopyranosyl-(1→4)-*O*-β-d-glucopyranosyl-(1→4)-*O*-β-d-glucopyranoside (10*R*) [14], 7,11-dimethyl-3-methylene-1,6-dodecadien-10,11-diol 10-*O*-β-d-glucopyranosyl-(1→4)-*O*-β-d-glucopyranoside (10*R*) [13], and icariside C**_4_** (10*R*) [17], while different from those of icariside C_1_ [17]. The assignments at C-8 and C-9 of icariside C_4_ and icariside C_1_ in the literature [17] should be interchanged. Based on the above evidence, **1** was defined as 3-(3′*E*-7′*R*,8′-dihydroxy-4′,8′-dimethyl-3′-nonenyl)-2,5-dihydro-1,1-dioxo-thiophen 7′-*O*-β-d-glucopyranosyl-(1→4)-*O*-β-d-glucopyranosyl-(1→4)-*O*-β-d-glucopyranoside.

Compound **2** was obtained as a white amorphous powder and its molecular formula C_27_H_46_O_14_S was established by HR-ESIMS at *m*/*z* 625.2515 [M − H]^−^ (calcd. for C_27_H_46_O_14_S 625.2530). Comparing the NMR and MS data of **2** with **1**, it was determined that **2** had the same aglycone as **1**. The ^13^C-NMR resonances of the sugar unit were identified by HSQC and further confirmed by HMBC experiments. The sugar moieties of **2** were the same as those of 7,11-dimethyl-3-methylene-1,6-dodecadien-10,11-diol 10-*O*-β–d-glucopyranosyl-(1→4)-*O*-β-d-glucopyranoside [13], as revealed by comparing the NMR data. The HMBC spectrum showed long-range correlations between H-1-Glc′ (δ 4.91) and C-10 (δ 90.0), H-1-Glc″ (δ 5.18) and C-4-Glc′ (δ 81.3) (Figure 2), which assigned the linkage of the sugar moiety. Therefore, **2** was defined as 3-(3′*E*-7′*R*,8′-dihydroxy-4′,8′-dimethyl-3′-nonenyl)-2,5-dihydro-1,1-dioxo-thiophen 7′-*O*-β-d-glucopyranosyl-(1→4)-*O*-β-d-glucopyranoside.

Compound **3** was obtained as a white amorphous powder and the molecular formula C_35_H_58_O_20_S was deduced by HR-ESIMS at *m*/*z* 829.3126 [M − H]^−^ (calcd. for C_35_H_58_O_20_S 829.3164). The NMR data of **3** were very similar to **1**, except for the presence of the CH_3_CO group. Furthermore, the CH_3_CO group located at the OH group of C-6 position in the inner Glc′, which turned into ester, was supported by the HMBC correlations between H-6-Glc′ (δ 5.13, 4.77) and C-CH_3_CO (δ 170.8). The sugar moieties of **3** were further assigned by the HSQC, HMBC, and ^1^H−^1^H COSY experiments. Therefore, **3** was defined as 3-(3′*E*-7′*R*,8′-dihydroxy-4′,8′-dimethyl-3′-nonenyl)-2,5-dihydro-1,1-dioxo-thiophen 7′-*O*-β-d-glucopyranosyl-6′-*O*-acetyl-(1→4)-*O*-β-d-glucopyranosyl-(1→4)-*O*-β-d-glucopyranoside.

Compound **1**–**3** were evaluated for cytotoxicity against L02 cells. All three compounds showed no cytotoxic activity at 100 μM.

## 3. Experimental

### 3.1. General Experimental Procedures

IR spectra, HR-ESIMS and NMR were recorded on a VERTEX 70 FT Infrared Spectrometer, Synapt MS (Waters Corporation, Milford, MA, USA) and Varian UNITYINOVA 600 spectrometer (600 MHz for ^1^H-NMR and 150 MHz for ^13^C-NMR, PaloAlto, CA, USA) in pyridine-*d*_5_ (Sigma-Aldrich, St. Louis, MO, USA), respectively. HPLC analyses were performed on Agilent 1100 system (Agilent Technologies, Santa Clara, CA, USA) equipped with a Silgreen C18 column (4.6 mm × 250 mm, ODS, 5 μm, Silgreen Co. Ltd., Beijing, China) and an Alltech 2000 evaporative light scattering detector (Temperature: 110 °C, Gas: 2.4 L/min, Alltech Corporation, Deerfield, IL, USA). Preparative HPLC was performed on an NP7000 module (Hanbon Co. Ltd., Huaian, China) equipped with a Shodex RID 102 detector (Showa Denko Group, Tokyo, Japan), and a Silgreen C18 column (20.0 mm × 250 mm, ODS, 5 μm, Silgreen Co. Ltd., Beijing, China). Semi-preparative HPLC was performed on a Waters 515 pump (Waters Corporation, Milford, MA, USA) equipped with a Shodex RID 101 detector (Showa Denko Group, Tokyo, Japan), using a Silgreen C18 column (10.0 mm × 250 mm, ODS, 5 μm, Silgreen Co. Ltd., Beijing, China). TLC was performed on silica gel GF254 plates (Qingdao Marine Chemical, Qingdao, China). Macroporous resin SP825 (Mitsubishi Chemicals, Tokyo, Japan), silica gel H (Qingdao Marine Chemical, Qingdao, China), and MCI gel (50 μm, Mitsubishi Chemicals, Tokyo, Japan) were applied to the performance of column chromatography.

### 3.2. Plant Material

The rhizomes of *T. tschonoskii* were collected from the Shennongjia of Hubei province, were identified by Professor Bao-Lin Guo (Institute of Medicinal Plant Development, Chinese Academy of Medical Sciences). A voucher specimen (No. 151010) was deposited in the author′s laboratory in the Beijing Institute of Radiation Medicine.

### 3.3. Extraction and Isolation

The rhizomes of *T. tschonoskii* (5 kg) were crushed and extracted with 50% aq. EtOH at reflux three times (40 L, 30 L, and 30 L, each for 2 h). The filtered solution was concentrated in vacuo. The supernatants were applied to a macroporous resin SP825 column, eluted with EtOH/H_2_O (5:95, 30:70, 50:50, 75:25 and 95:10, *v*/*v*) to yield five factions (Fr. A−Fr. E). Fr. C (120 g) was subjected to silica-gel CC with a gradient mixture of CHCl_3_:MeOH:H_2_O (5:1:0.01, 65:25:4, and 2:1:0.01) as the eluent, and five subfractions were obtained (Fr. C-1−Fr. C-5). Fr. C-3 (35 g) was further subjected to MCI gel CC with a gradient mixture of acetone/H_2_O (10:90, 15:85, 20:80, 30:70 and 50:50, *v*/*v*) as the eluent. As a result, a total of 30 fractions were collected (Fr. C-3-1−Fr. C-3-30). Fr. C-3-6 was purified by preparative HPLC with ACN/H_2_O (20:80, *v*/*v*) to obtain seven fractions (Fr. C-3-6-1−Fr. C-3-6-7). Fr. C-3-6-4 underwent semi-preparative HPLC with acetone/H_2_O (25:75, *v*/*v*) to yield **1** (55 mg) and **2** (12 mg). Fr. C-3-12 was purified by preparative HPLC with ACN/H_2_O (22:78, *v*/*v*) to yield five fractions (Fr. C-3-12-1~Fr. C-3-12-5). Fr. C-1-12-5 was purified by semi-preparative HPLC with ACN/H_2_O (20:80, *v*/*v*) to yield **3** (15 mg).

*3-(3′E-7′R,8′-Dihydroxy-4′,8′-dimethyl-3′-nonenyl)-2,5-dihydro-1,1-dioxo-thiophen 7′-O-β-d-glucopyranosyl-(1→4)-O-β-d-glucopyranosyl-(1→4)-O-β-d-glucopyranoside* (**1**): White amorphous power; ${\left[\alpha \right]}_{D}^{20}$ −10.5° (*c* 0.085, MeOH); IR (KBr) ν_max_ 3408, 2970, 2927, 1642, 1382, 1306, 1236, 1159, 1072, 1026; ^1^H-NMR and ^13^C-NMR spectroscopic data, see Table 1; HR-ESI-MS (positive) *m*/*z* 789.3241 [M + H]^+^ (calcd. for C_33_H_55_O_19_S 789.3215), 811.3036 [M + Na]^+^, 627.2696 [M + H − Glc]^+^, 465.2148 [M + H − Glc − Glc]^+^, 303.1644 [M + H − Glc − Glc − Glc]^+^.

*3-(3′E-7′R,8′-Dihydroxy-4′,8′-dimethyl-3′-nonenyl)-2,5-dihydro-1,1-dioxo-thiophen 7′-O-β-d-glucopyranosyl-(1→4)-O-β-d-glucopyranoside* (**2**): White amorphous power; ${\left[\alpha \right]}_{D}^{20}$ −10.3° (*c* 0.082, MeOH); IR (KBr) ν_max_ 3433, 2928, 1642, 1383, 1306, 1237, 1074, 1041; ^1^H-NMR and ^13^C-NMR spectroscopic data, see Table 1; HR-ESIMS (positive) *m*/*z* 627.2679 [M + H]^+^ (calcd. for C_27_H_47_O_14_S 627.2687), 649.2488 [M + Na]^+^, 465.2147 [M + H − Glc]^+^, 303.1637 [M + H − Glc − Glc]^+^.

*3-(3′E-7′R,8′-Dihydroxy-4′,8′-dimethyl-3′-nonenyl)-2,5-dihydro-1,1-dioxo-thiophen 7′-O-β-d-glucopyranosyl-6′-O-acetyl-(1→4)-O-β-d-glucopyranosyl-(1→4)-O-β-d-glucopyranoside* (**3**): White amorphous power; ${\left[\alpha \right]}_{D}^{20}$ −12.5° (*c* 0.088, MeOH); IR (KBr) ν_max_ 3428, 2970, 2926, 1736, 1644, 1383, 1307, 1239, 1161, 1072, 1029; ^1^H-NMR and ^13^C-NMR spectroscopic data, see Table 1; HR-ESIMS (positive) *m*/*z* 831.3329 [M + H]^+^ (calcd. for C_35_H_59_O_20_S 831.3320), 853.3165 [M + Na]^+^, 669.2820 [M + H − Glc]^+^, 507.2261 [M + H − Glc − Glc]^+^, 303.1612 [M + H − Glc − Glc − (Ac-Glc)]^+^.

### 3.4. Acid Hydrolysis and GC-MS Analysis

Compound **1** (1.4 mg), **2** (1.6 mg), and **3** (1 mg) were hydrolyzed with 2 N aq. CF_3_COOH (5 mL) for 5 h at 95 °C, respectively. After extraction with CH_2_Cl_2_ (3 × 5 mL), the aq. layer was repeatedly evaporated to dryness with EtOH until neutral, and then analyzed by TLC over silica gel (CHCl_3_:MeOH:H_2_O, 8:5:1) by comparison with authentic samples. Furthermore, the residue of sugars was dissolved in anhydrous pyridine (2 mL), and l-cysteine methyl ester hydrochloride (3 mg) was added. The mixture was stirred at 60 °C for 1 h, then 3 mL of HMDS-TMCS (hexamethyldisilazane:trimethylchlorosilane, 2:1) was added, and the mixture was stirred at 60 °C for 30 min. The precipitate was centrifuged off, and the supernatant was analyzed by GC-MS (Agilent Technologies 5977A MSD). The absolute configurations were determined by comparing the retention times with derivatives prepared in a similar way from standard d-glucose (Sigma-Aldrich). Identification of d-glucose was carried out for compounds **1**–**3**, giving two peaks at 3.75 min and 4.15 min which were two silylated derivatives (Supplementary Material, Figure S25).

### 3.5. Cytotoxicity Assay

The cytotoxic activity was measured by MTT assay [18]. L02 Cells were seeded in 96-well plates and treated 24 h later with various (100 μΜ, 50 μΜ, 25 μΜ, 12.5 μΜ, 6.25 μΜ, 3.125 μΜ) concentrations of compounds **1**–**3**. After 24 h of incubation, MTT was added to all wells. Plates were incubated for another 24 h, and cell viability was measured by observing absorbance at 492 nm.

## 4. Conclusions

In conclusion, three new sesquiterpene glycosides were isolated from the rhizomes of *T. tschonoskii*. Their structures were elucidated by extensive analysis of spectroscopic methods including 1D and 2D NMR experiments (HSQC, HMBC, ^1^H–^1^H COSY), IR, and HR-ESI-MS. The aglycone of the compounds found in this study was a rare aglycone which contains a sulfonyl between C-1 and C-15 positions. Compounds **1**–**3** were investigated for their cytotoxic activity against L02 cells, and no obvious cytotoxic activity was found.

## Figures and Tables

**Table 1:** ^1^H-NMR, ^13^C-NMR data for compounds **1**–**3**.

No.	1		2		3	
	δ_C_	δ_H_	δ_C_	δ_H_	δ_C_	δ_H_
2	58.0	3.85 (2H, m)	58.0	3.85 (2H, m)	58.0	3.85 (2H, m)
3	138.7		138.7		138.7	
4	117.8	5.58 (1H, m)	117.8	5.58 (1H, m)	117.8	5.58 (1H, m)
5	57.4	3.89 (2H, m)	57.4	3.89 (2H, m)	57.4	3.89 (2H, m)
1′	33.1	2.07 (2H, m)	33.1	2.07 (2H, m)	33.1	2.07 (2H, m)
2′	25.6	2.09 (2H, m)	25.6	2.09 (2H, m)	25.6	2.09 (2H, m)
3′	123.6	5.34 (1H, br t, *J* = 7.2Hz)	123.6	5.34 (1H, br t, *J* = 7.2Hz)	123.6	5.34 (1H, br t, *J* = 7.2Hz)
4′	136.8		136.8		136.8	
5′	36.2	2.74 (1H, m)	36.1	2.74 (1H, m)	36.1	2.74 (1H, m)
		2.50 (1H, m)		2.50 (1H, m)		2.50 (1H, m)
6′	30.8	1.84 (1H, m)	30.8	1.84 (1H, m)	30.8	1.84 (1H, m)
		1.76 (1H, m)		1.76 (1H, m)		1.76 (1H, m)
7′	90.0	3.75 (1H, dd, *J* = 1.5, 9.5Hz)	90.0	3.75 (1H, dd, *J* = 1.5, 9.5Hz)	90.4	3.75 (1H, dd, *J* = 1.5, 9.5Hz)
8′	71.9		71.9		71.9	
9′	25.3	1.37 (3H, s)	25.3	1.37 (3H, s)	25.5	1.37 (3H, s)
8′-CH_3_	26.8	1.32 (3H, s)	26.8	1.32 (3H, s)	26.7	1.32 (3H, s)
4′-CH_3_	16.1	1.61 (3H, s)	16.1	1.61 (3H, s)	16.1	1.61 (3H, s)
						
Glc-1′	105.7	4.89 (1H, d, *J* = 7.8 Hz)	105.7	4.91 (1H, d, *J* = 7.8Hz)	105.6	4.86 (1H, d, *J* = 7.8 Hz)
Glc-2′	74.3	4.05 (1H, o)	74.8	4.05 (1H, o)	71.9	4.23 (1H, o)
Glc-3′	76.5	4.22 (1H, o)	78.5	4.00 (1H, o)	76.5	4.22 (1H, o)
Glc-4′	80.9	4.28 (1H, o)	81.3	4.31 (1H, o)	81.3	4.23 (1H, o)
Glc-5′	76.4	3.95 (1H, o)	76.8	4.28 (1H, o)	76.8	4.00 (1H, o)
Glc-6′	61.9	4.47 (1H, o)	62.0	4.50 (2H, o)	62.1	4.58 (1H, o)
		4.47 (1H, o)				4.44 (1H, o)
Glc-1′′	105.0	5.12 (1H, d, *J* = 7.8 Hz)	105.0	5.18 (1H, d, *J* = 7.8Hz)	105.0	5.00 (1H, d, *J* = 7.8 Hz)
Glc-2′′	74.7	4.05 (1H, o)	75.0	4.05 (1H, o)	74.2	4.03 (1H, o)
Glc-3′′	76.7	4.22 (1H, o)	78.3	4.17 (1H, o)	78.5	4.01 (1H, o)
Glc-4′′	80.9	4.28 (1H, o)	71.6	4.17 (1H, o)	81.5	4.00 (1H, o)
Glc-5′′	78.5	3.96 (1H, o)	76.6	3.95 (1H, o)	73.2	4.13 (1H, o)
Glc-6′′	61.8	4.49 (1H, o)	62.5	4.52 (1H, dd, *J* = 2.4, 11.4 Hz)	64.3	5.13 (1H, dd, *J* = 2.4, 11.4 Hz)
		4.49 (1H, o)		4.29 (1H, o)		4.77 (1H, o)
Glc-1′′′	104.5	5.15 (1H, d, *J* = 7.8 Hz)			104.7	5.13 (1H, d, *J* = 7.8 Hz)
Glc-2′′′	75.0	4.05 (1H, o)			74.8	4.05 (1H, o)
Glc-3′′′	76.6	4.17 (1H, o)			76.6	3.95 (1H, o)
Glc-4′′′	71.5	4.17 (1H, o)			71.6	4.15 (1H, o)
Glc-5′′′	78.2	3.95 (1H, o)			78.2	4.17 (1H, o)
Glc-6′′′	62.5	4.52 (1H, dd, *J* = 2.4, 11.4 Hz)			62.5	4.52 (1H, dd, *J* = 2.4, 11.4 Hz)
		4.29 (1H, o)				4.28 (1H, o)
CH3CO					20.8	2.08 (3H, s)
CH3CO					170.8	

δ in C_5_D_5_N, in ppm from tetramethylsilane (TMS), ^1^H-NMR at 600 MHz, ^13^C-NMR at 150 MHz; o: overlapped with other signals; m: multiplet signals.

## Supplementary Materials

The supplementary materials are available online.
