# Supplementary material for: Three New Sesquiterpene Glycosides from the Rhizomes of Trillium tschonoskii
Source: Molecules. 2017 Aug 2;22(8):1283. doi: 10.3390/molecules22081283 (PMC6152036; doi:10.3390/molecules22081283)
Supplement: Supplementary file 1 [file molecules-22-01283-s001.pdf]

Supplementary information

List of contents

Fig. 1S The mass (negetive) chromatography of compound **1**

Fig. 2S The mass (positive) chromatography of compound **1**

Fig. 3S The mass (negetive) chromatography of compound **2**

Fig. 4S The mass (positive) chromatography of compound **2**

Fig. 5S The mass (negetive) chromatography of compound **3**

Fig. 6S The mass (positive) chromatography of compound **3**

Fig. 7S ^{1}H NMR spectrum of compound **1**

Fig. 8S ^{13}C NMR spectrum of compound **1**

Fig. 9S HSQC spectrum of compound **1**

Fig. 10S ^{1}H-^{1}H COSY spectrum of compound **1**

Fig. 11S HMBC spectrum of compound **1**

Fig. 12S ^{1}H NMR spectrum of compound **2**

Fig. 13S ^{13}C NMR spectrum of compound **2**

Fig. 14S HSQC spectrum of compound **2**

Fig. 15S ^{1}H-^{1}H COSY spectrum of compound **2**

Fig. 16S HMBC spectrum of compound **2**

Fig. 17S ^{1}H NMR spectrum of compound **3**

Fig. 18S ^{13}C NMR spectrum of compound **3**

Fig. 19S HSQC spectrum of compound **3**

Fig. 20S ^{1}H-^{1}H COSY spectrum of compound **3**

Fig. 21S HMBC spectrum of compound **3**

Fig. 22S IR spectrum of compound **1**

Fig. 23S IR spectrum of compound **2**

Fig. 24S IR spectrum of compound **3**

Fig. 25S Chromatography of D-glucose and the acid hydrolysis products of compounds **1-3** separated by GC-MS

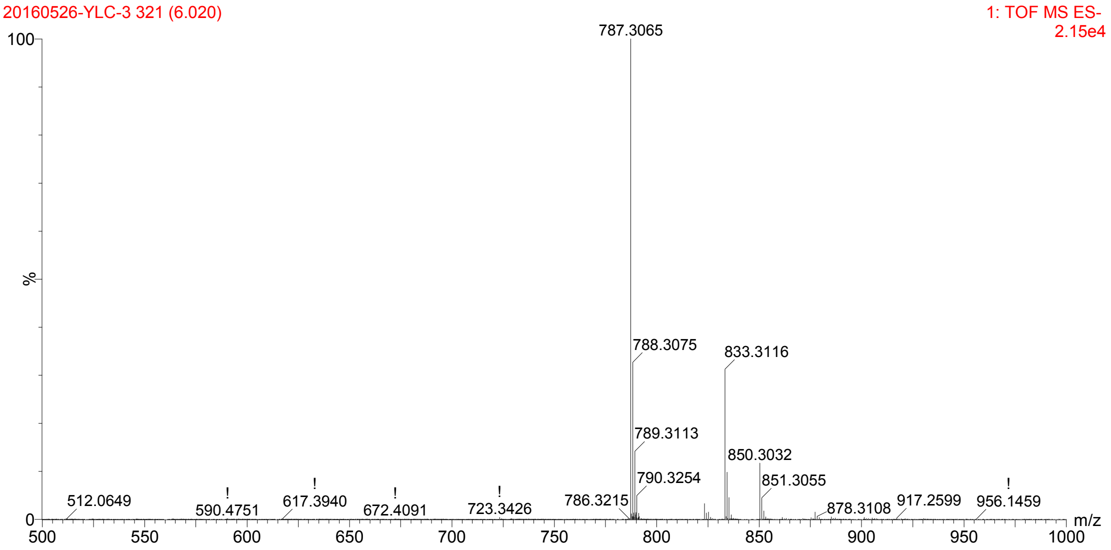

Fig. 1S The mass (negetive) chromatography of compound **1**

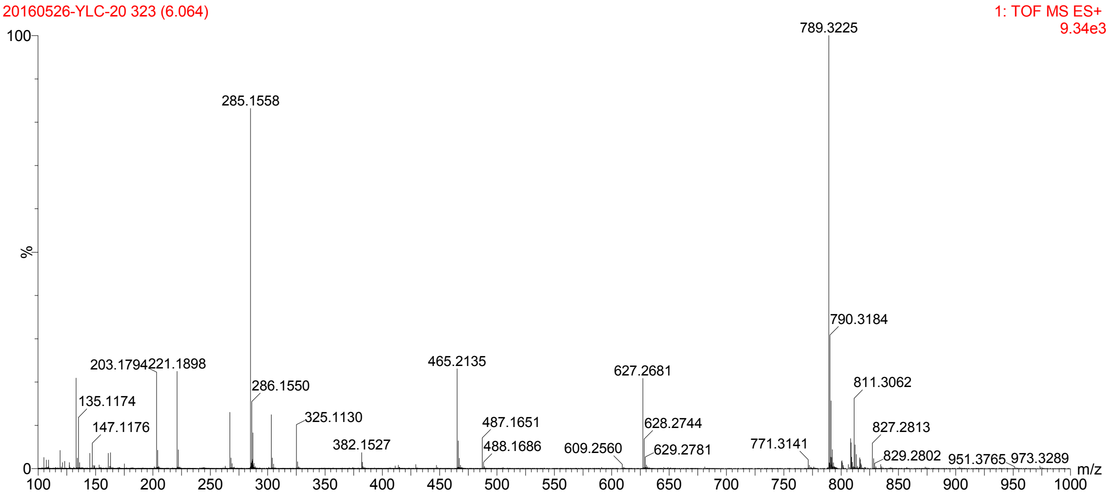

Fig. 2S The mass (positive) chromatography of compound **1**

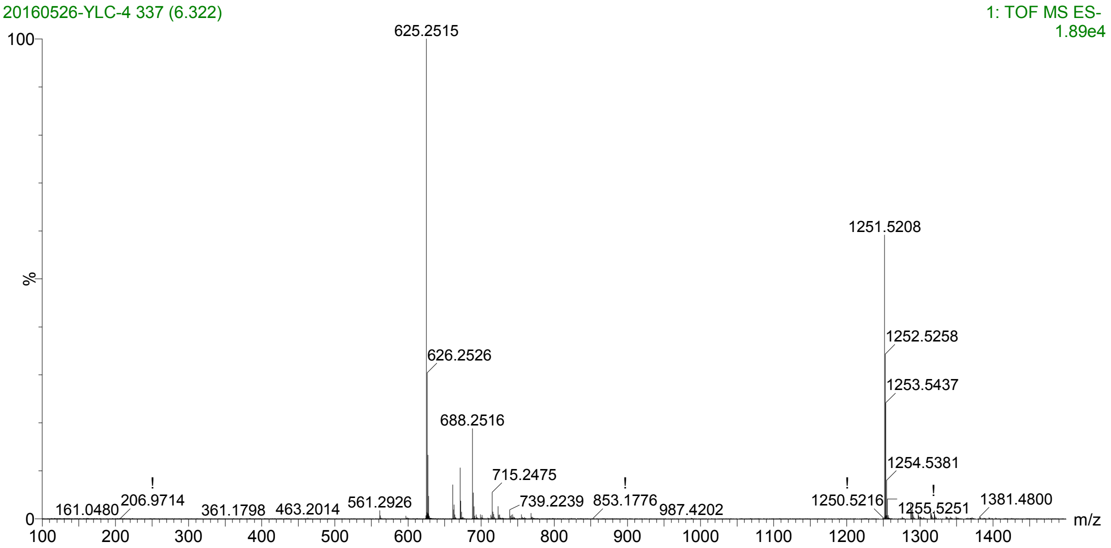

Fig. 3S The mass (negetive) chromatography of compound **2**

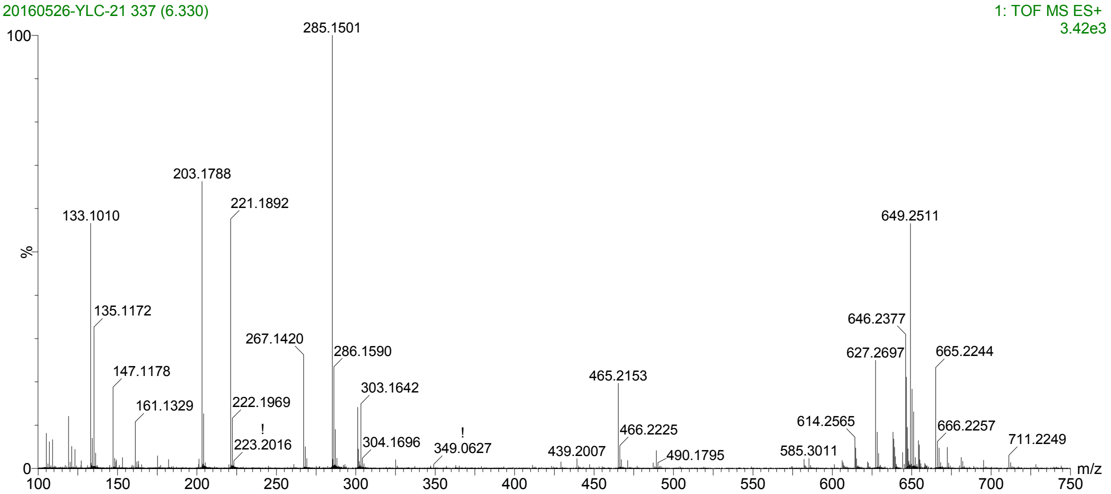

Fig. 4S The mass (positive) chromatography of compound **2**

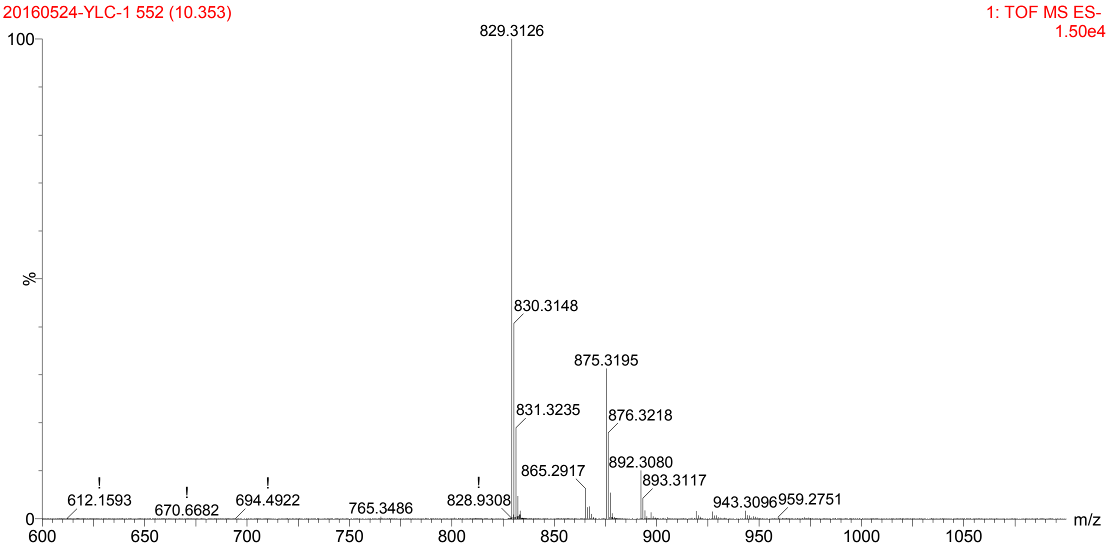

Fig. 5S The mass (negetive) chromatography of compound **3**

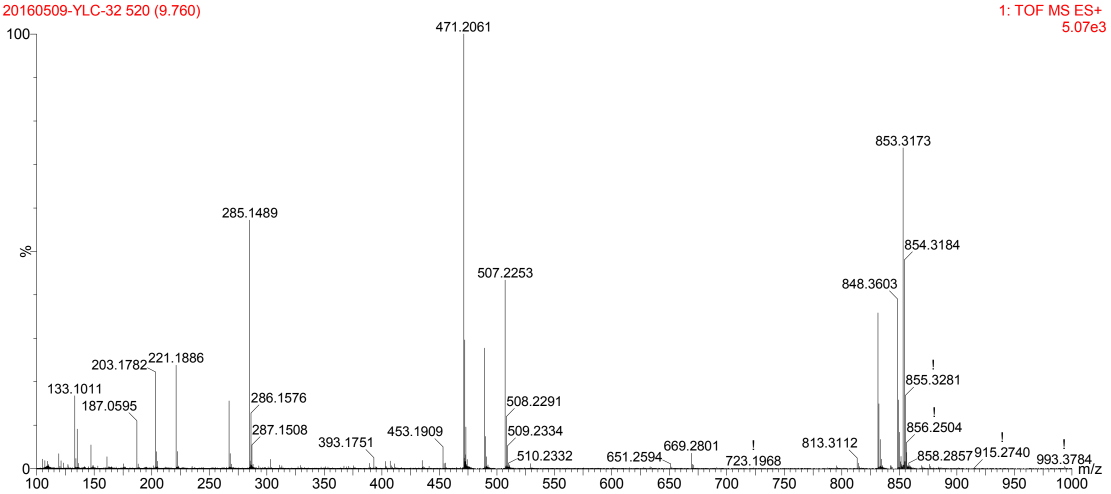

Fig. 6S The mass (positive) chromatography of compound **3**

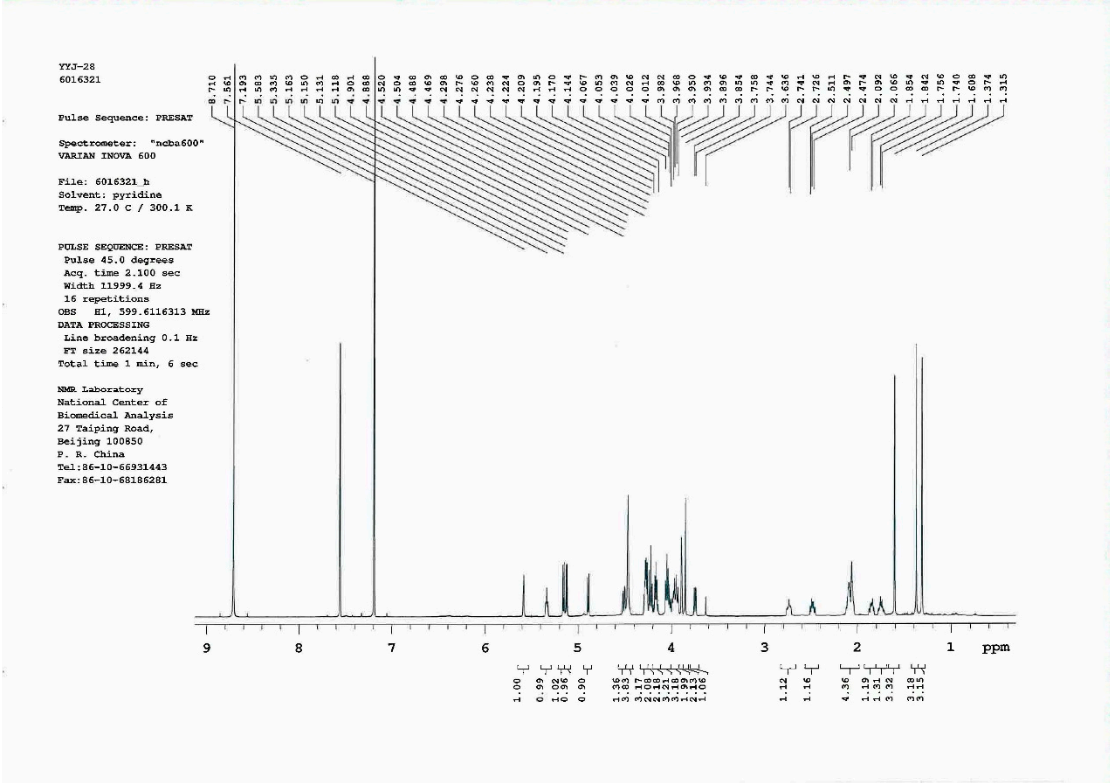

Fig. 7S ^{1}H NMR spectrum of compound **1**

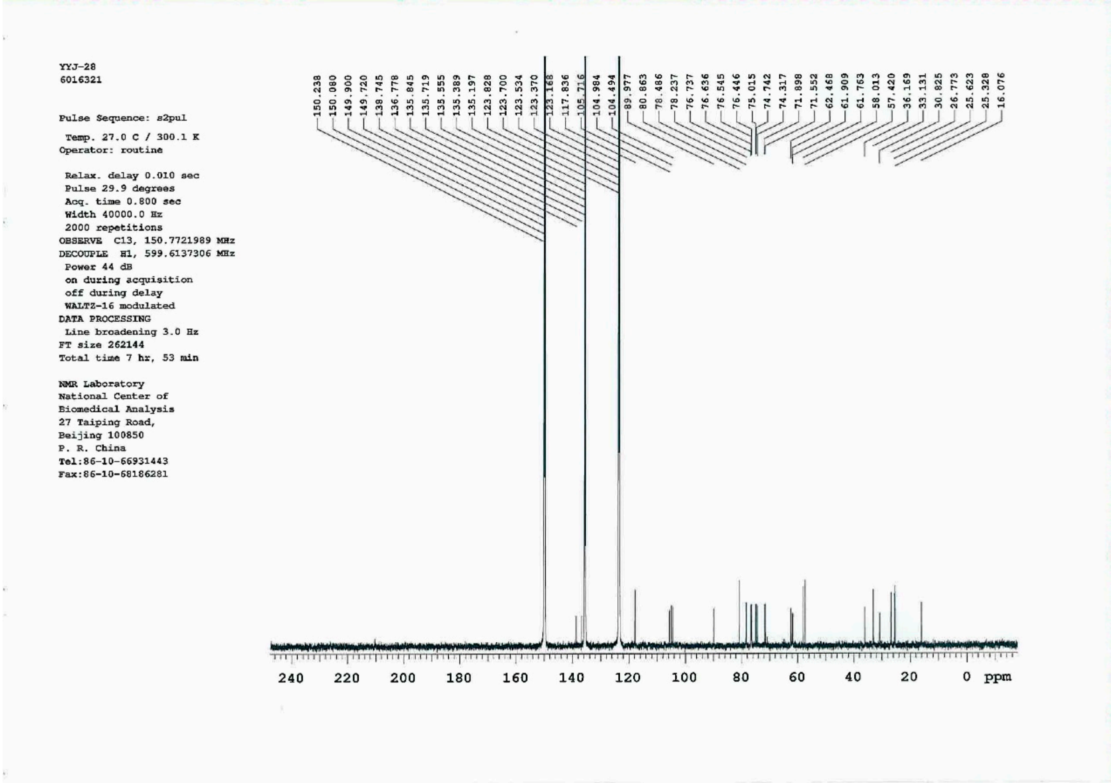

Fig. 8S ^{13}C NMR spectrum of compound **1**

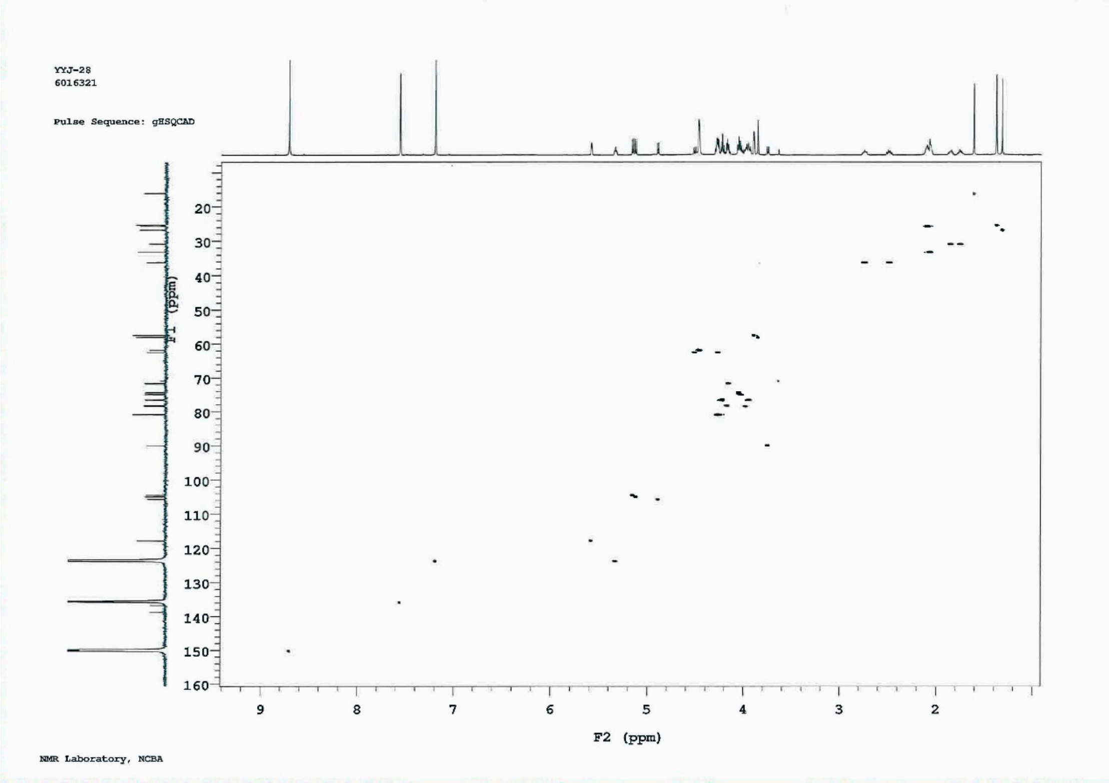

Fig. 9S HSQC spectrum of compound **1**

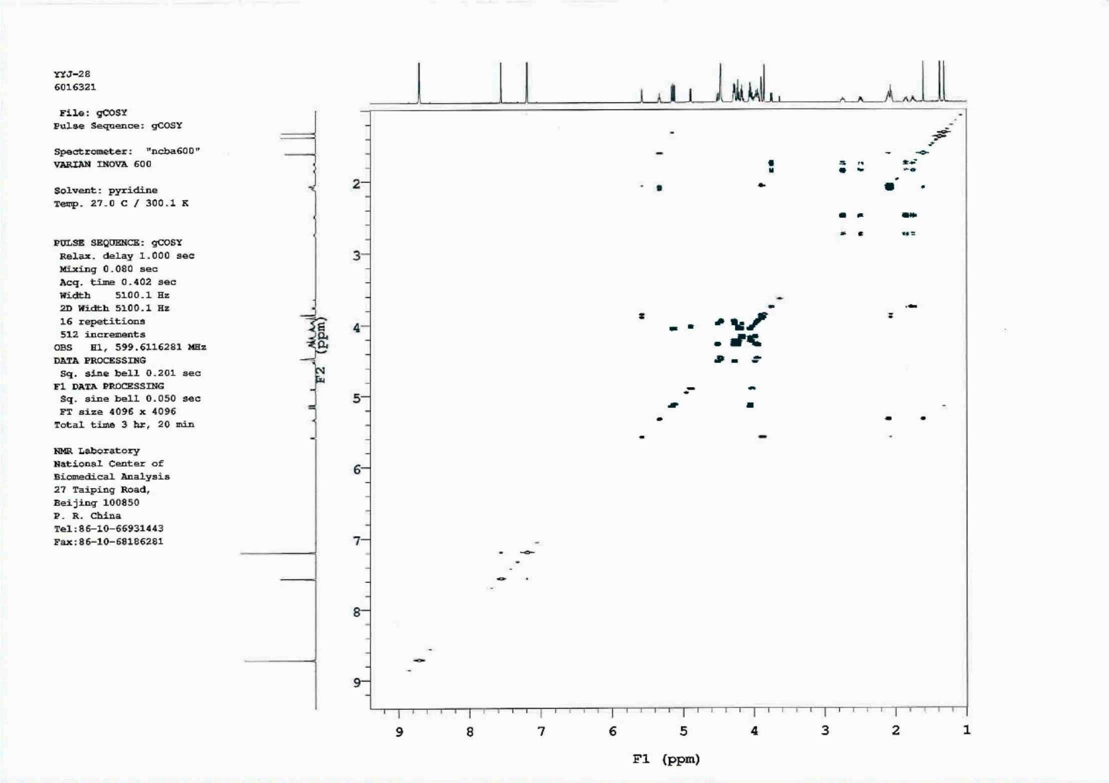

Fig. 10S ^{1}H-^{1}H COSY spectrum of compound **1**

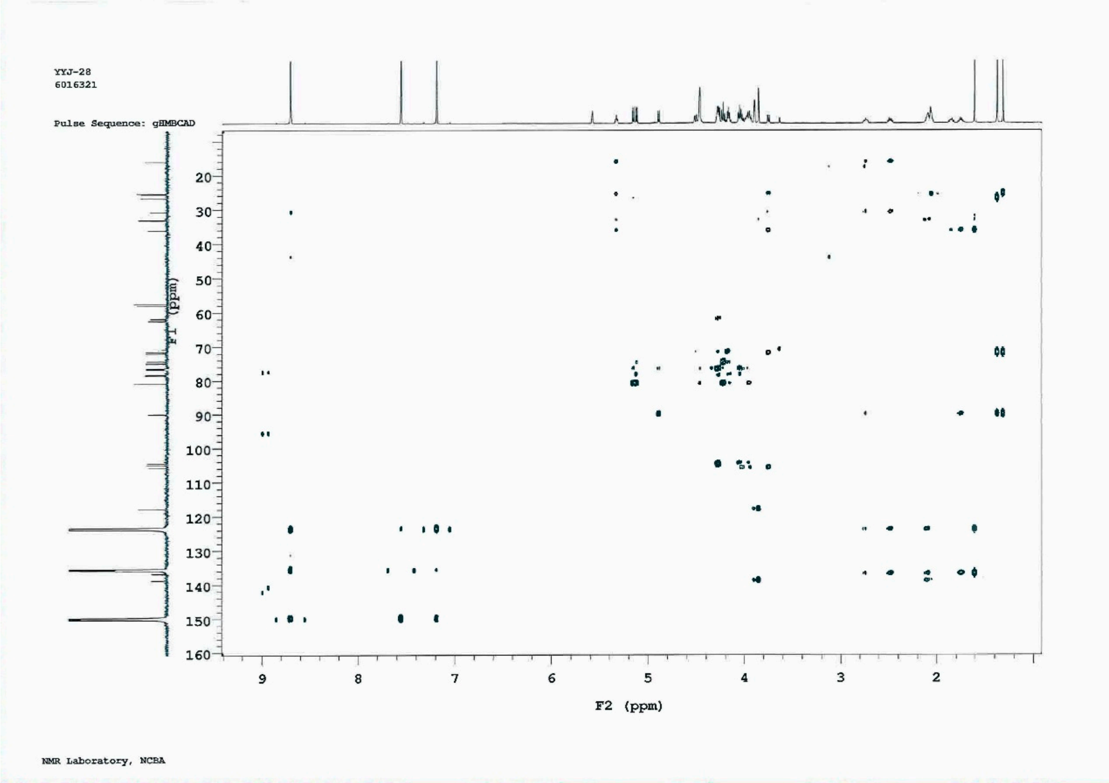

Fig. 11S HMBC spectrum of compound **1**

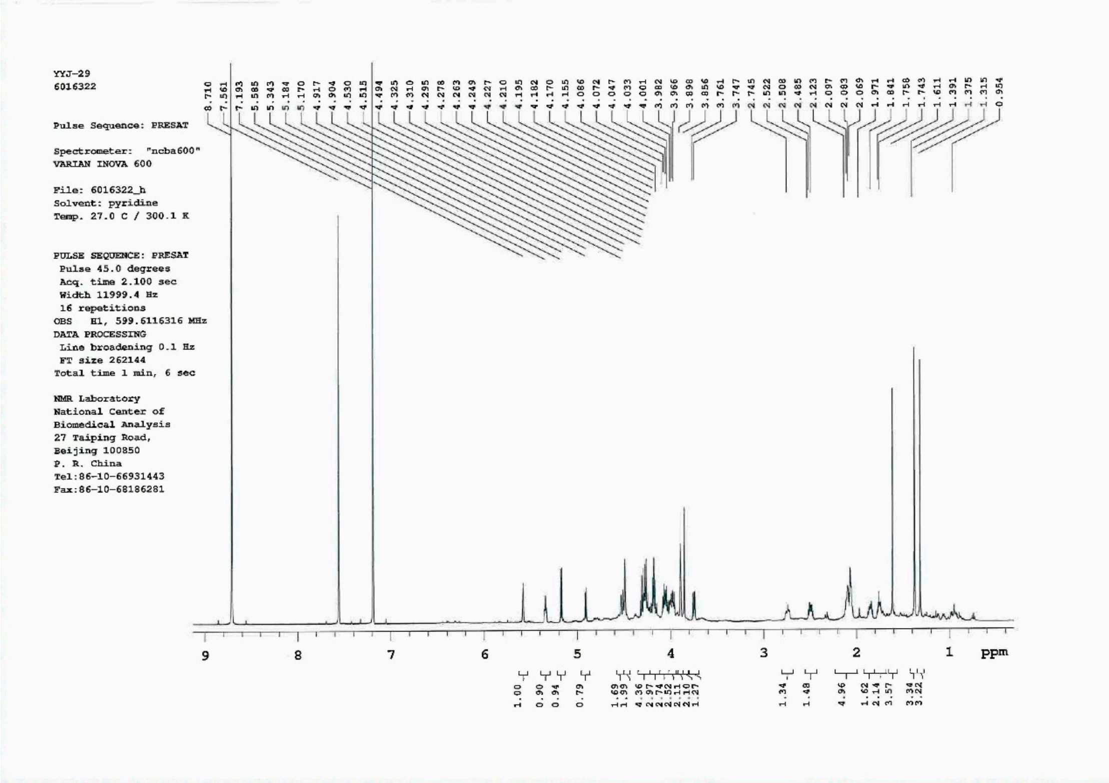

Fig. 12S ^{1}H NMR spectrum of compound **2**

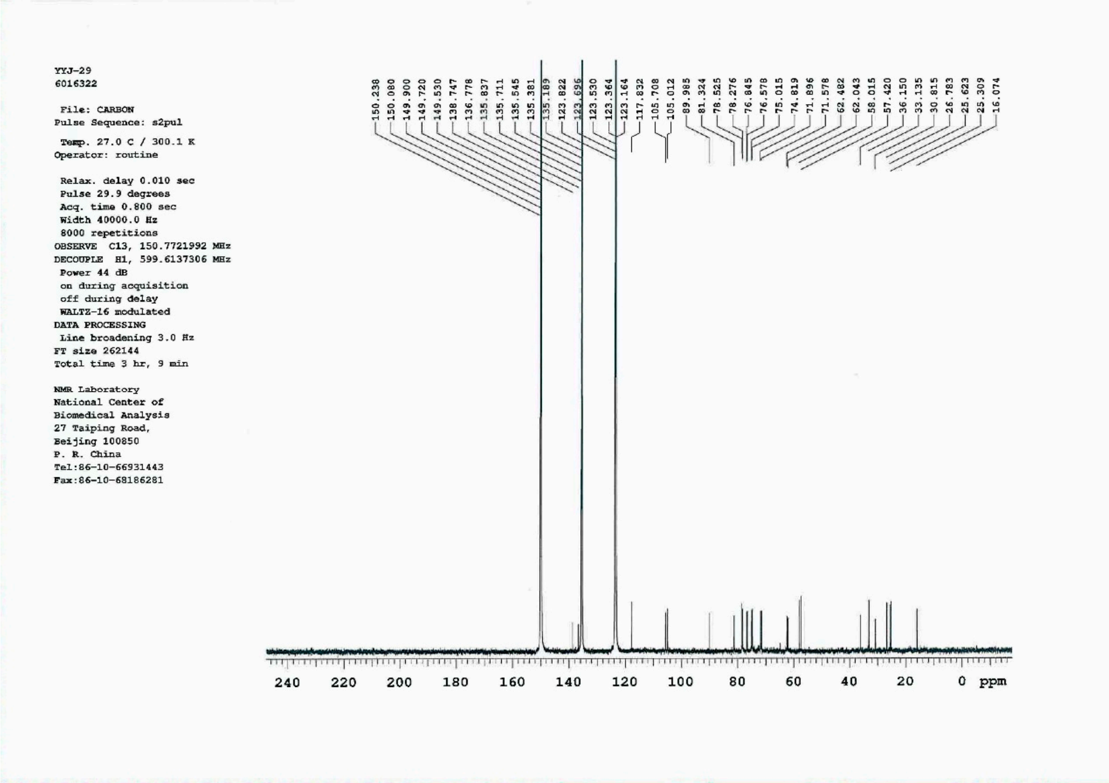

Fig. 13S ^{13}C NMR spectrum of compound **2**

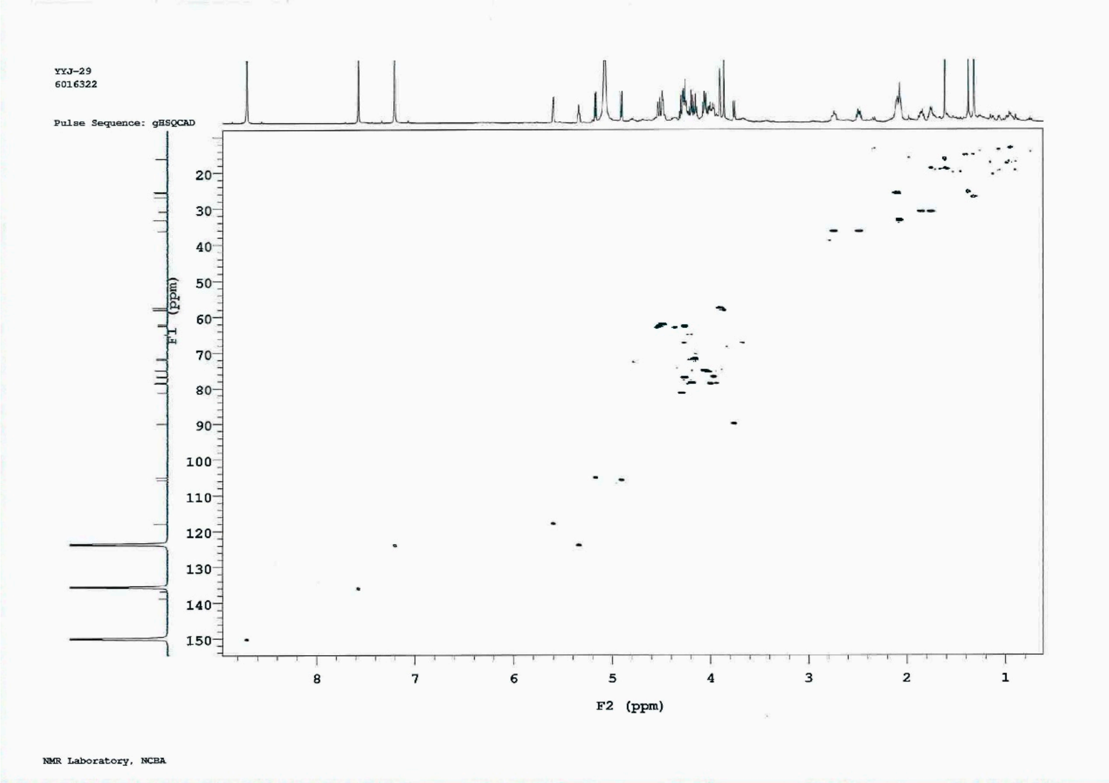

Fig. 14S HSQC spectrum of compound **2**

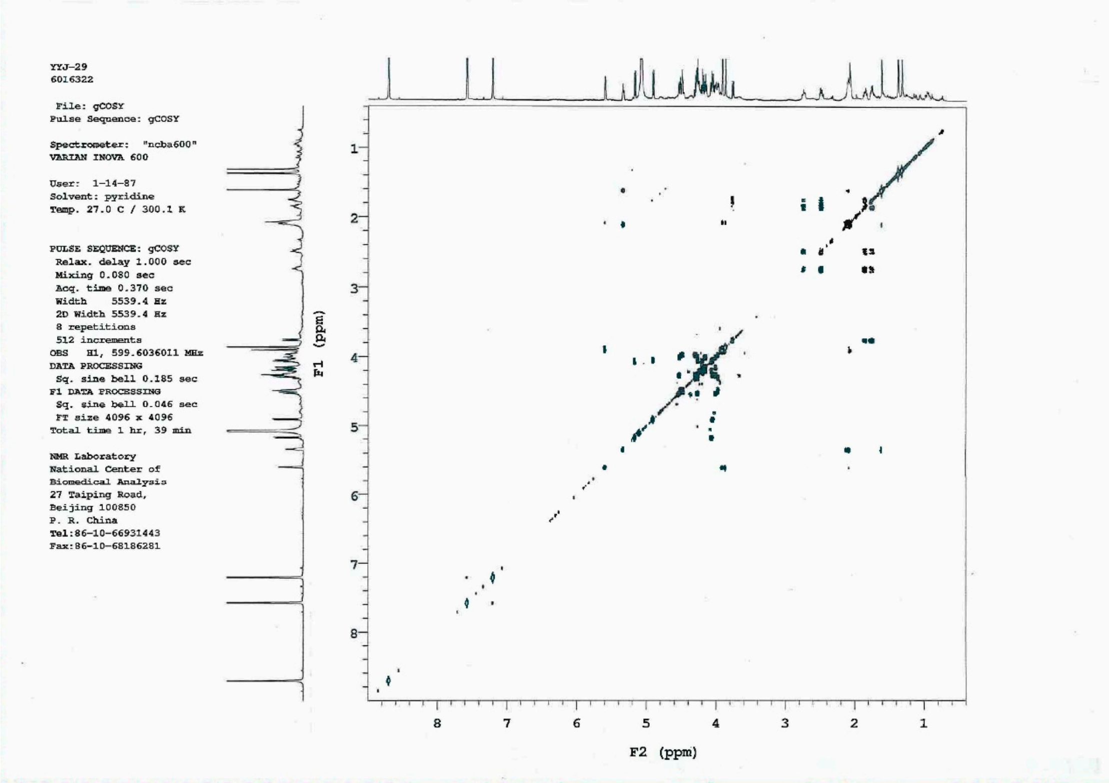

Fig. 15S ^{1}H-^{1}H COSY spectrum of compound **2**

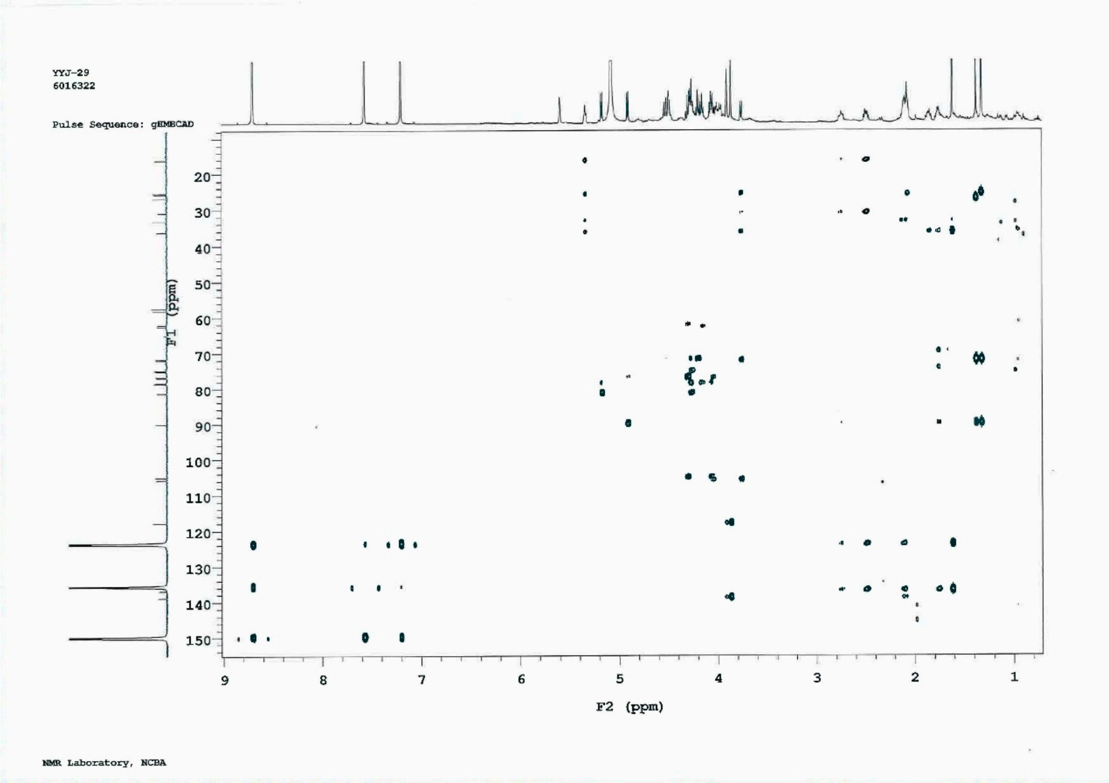

Fig. 16S HMBC spectrum of compound **2**

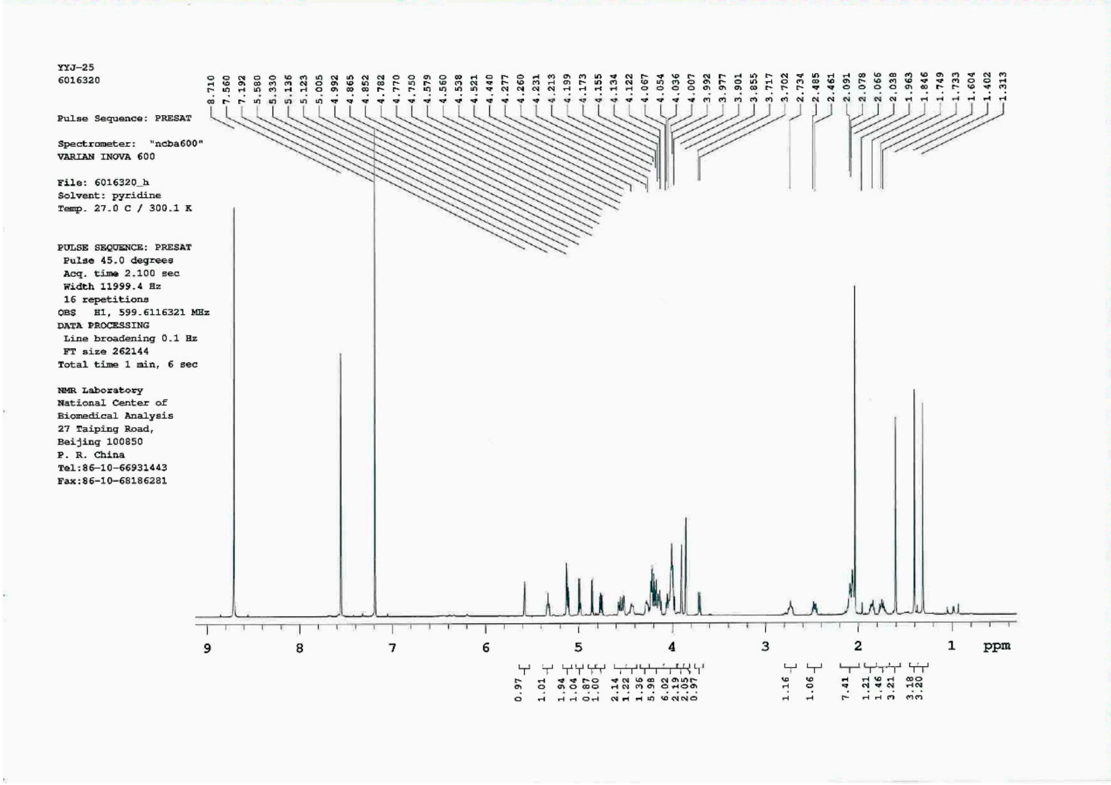

Fig. 17S ^{1}H NMR spectrum of compound **3**

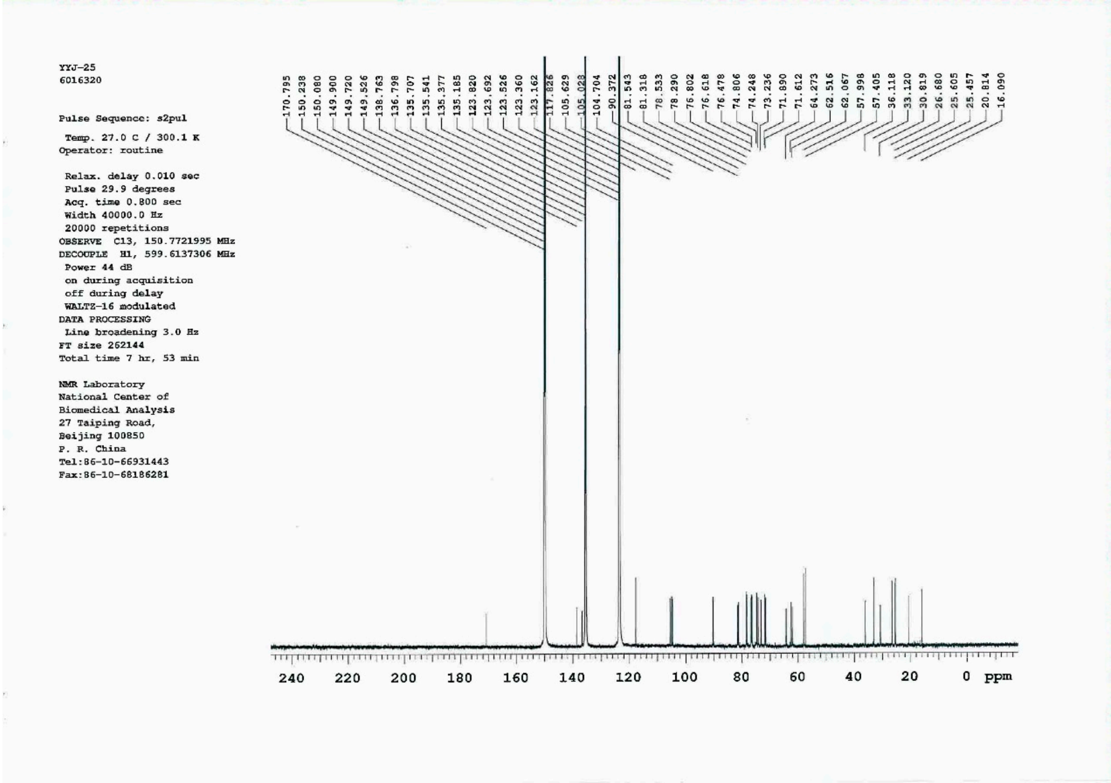

Fig. 18S ^{13}C NMR spectrum of compound **3**

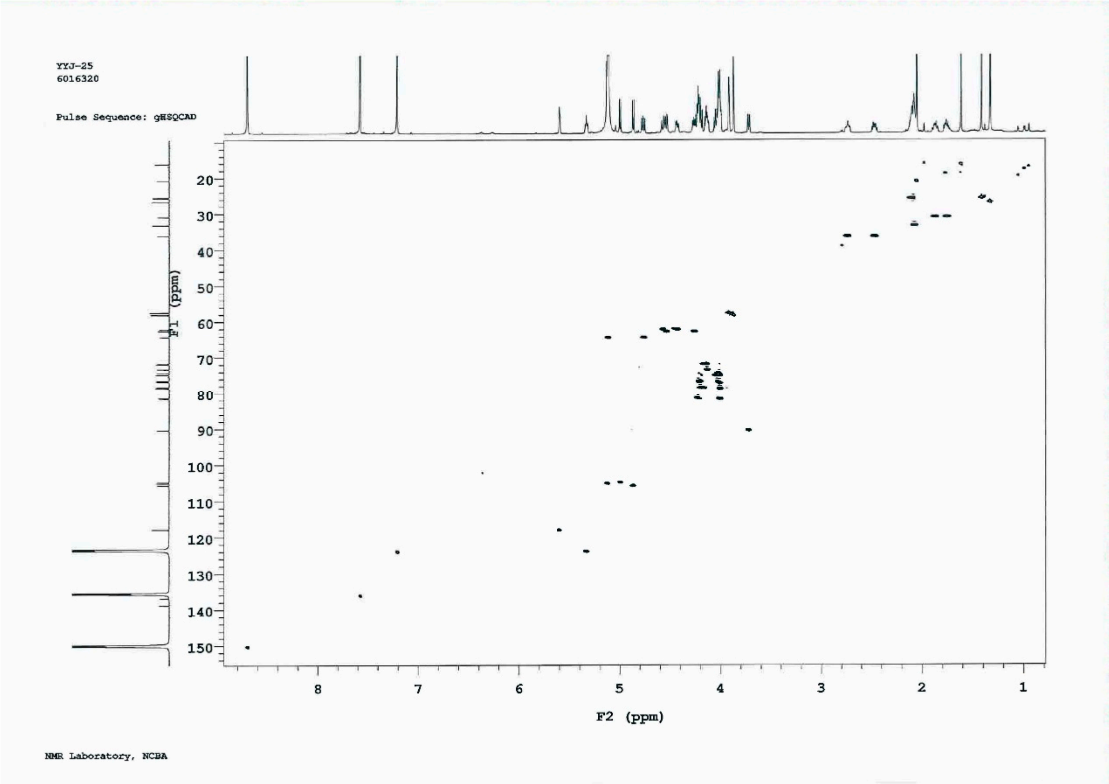

Fig. 19S HSQC spectrum of compound **3**

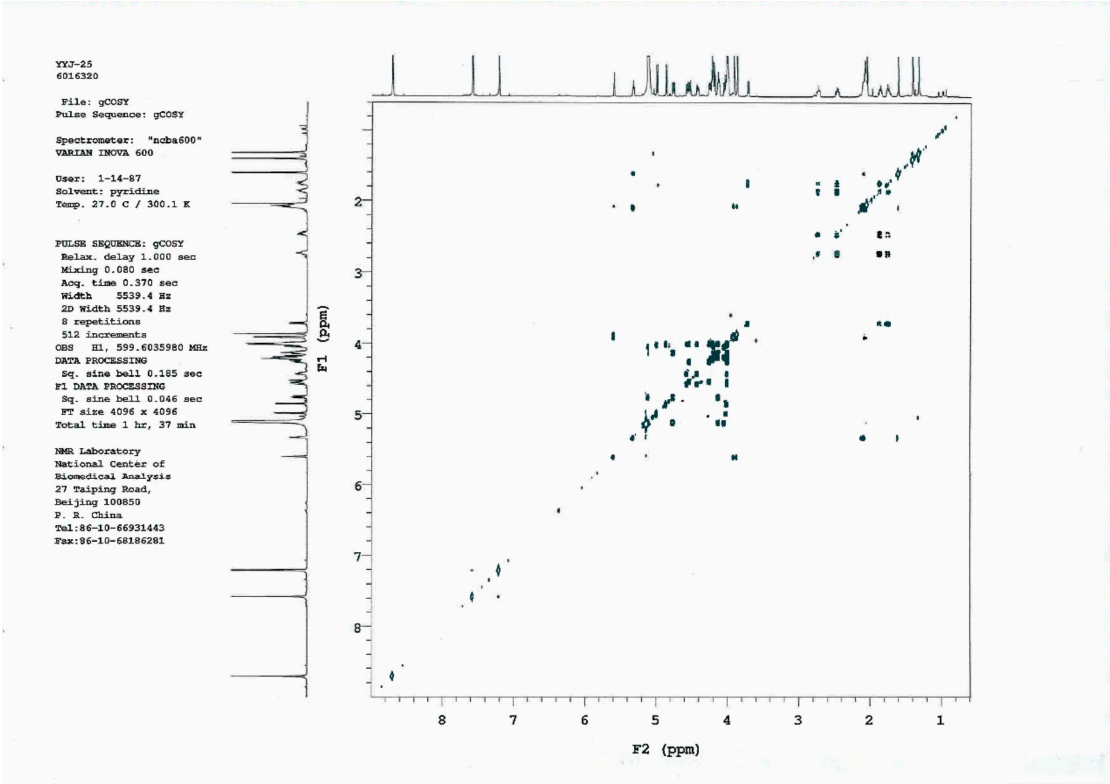

Fig. 20S ^{1}H-^{1}H COSY spectrum of compound **3**

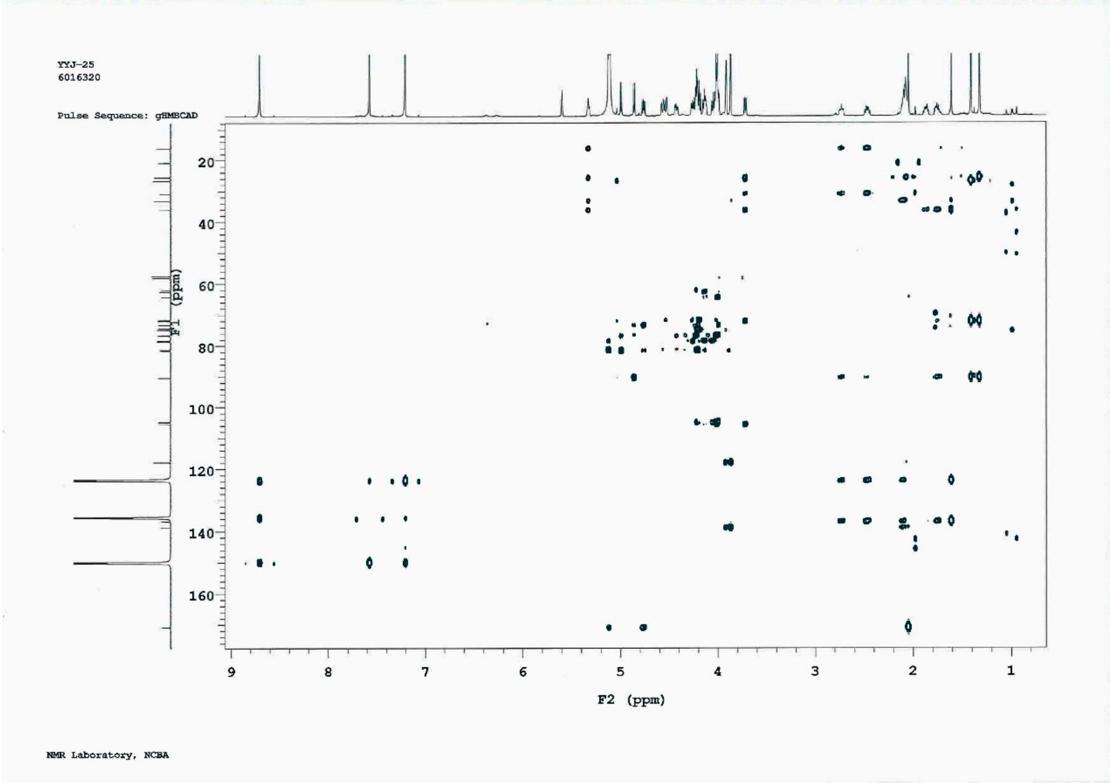

Fig. 21S HMBC spectrum of compound **3**

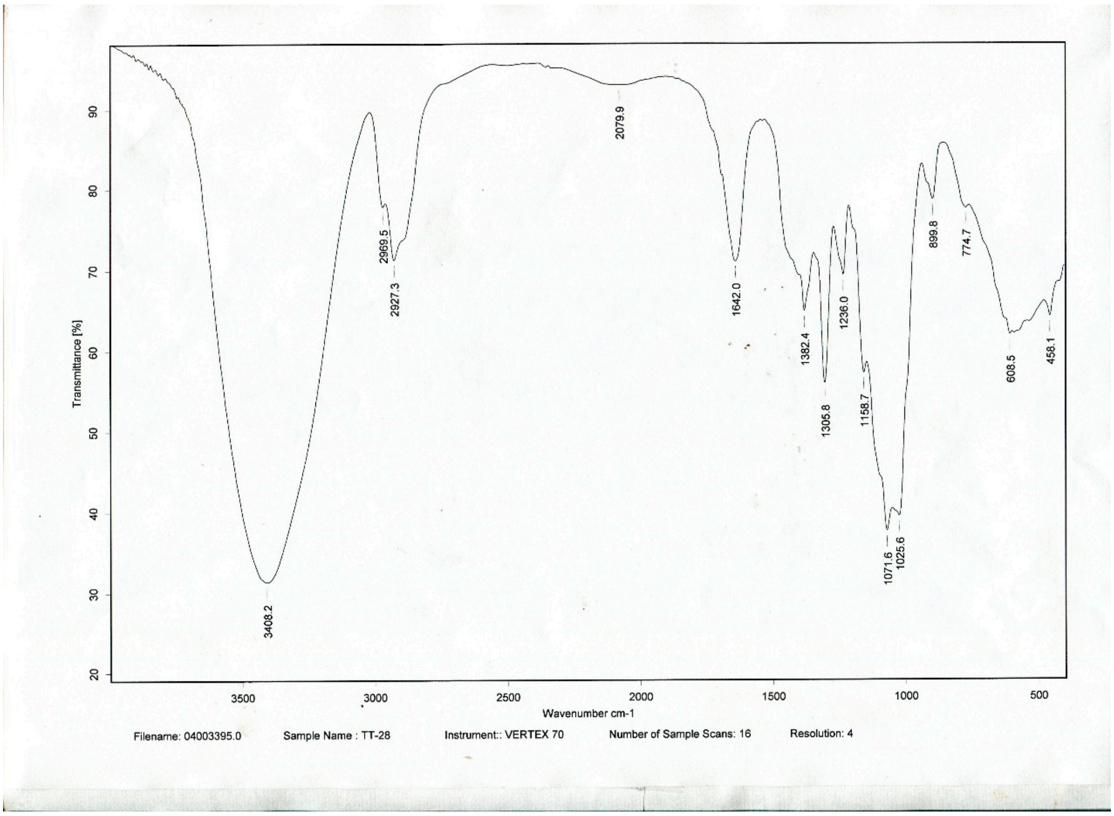

Fig. 22S IR spectrum of compound **1**

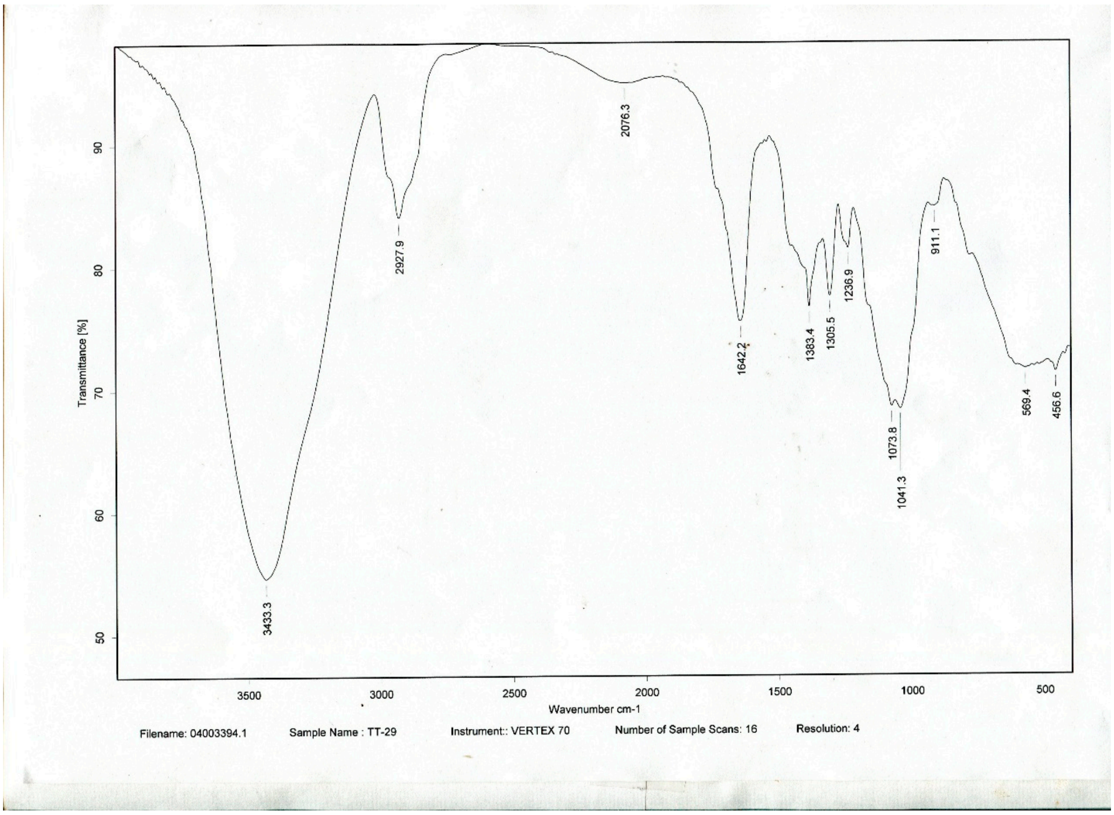

Fig. 23S IR spectrum of compound **2**

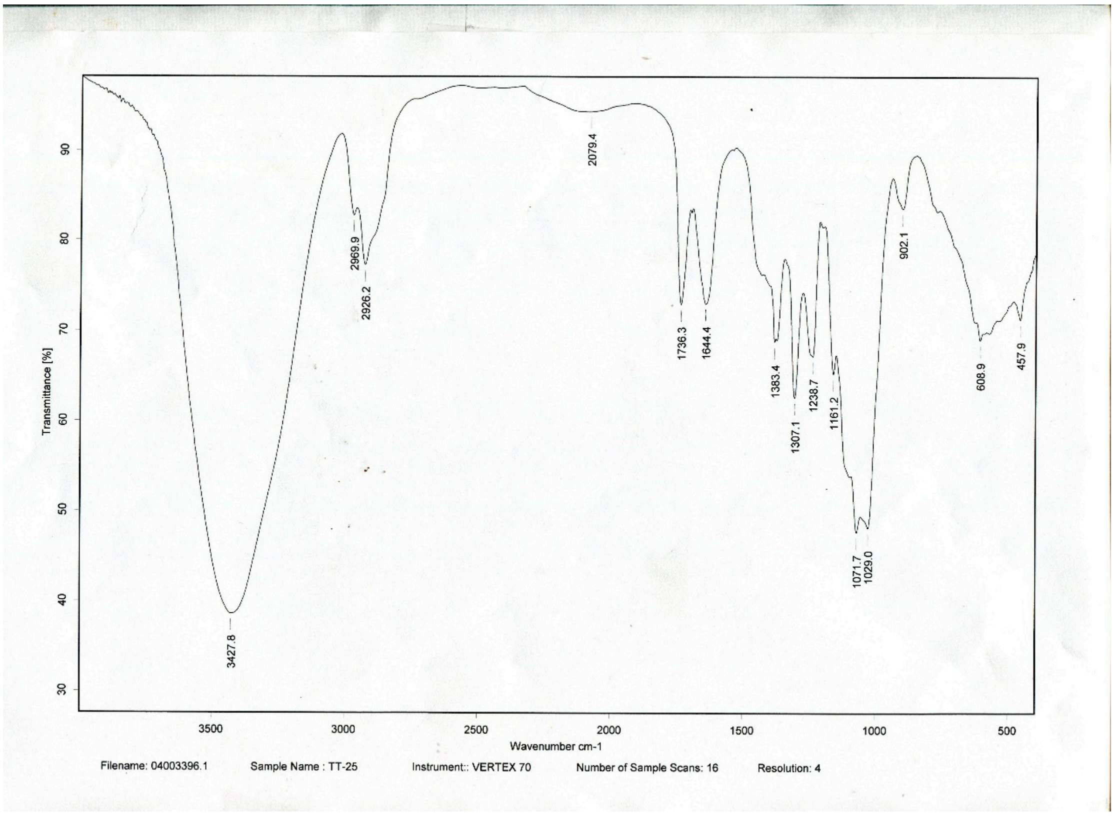

Fig. 24S IR spectrum of compound **3**

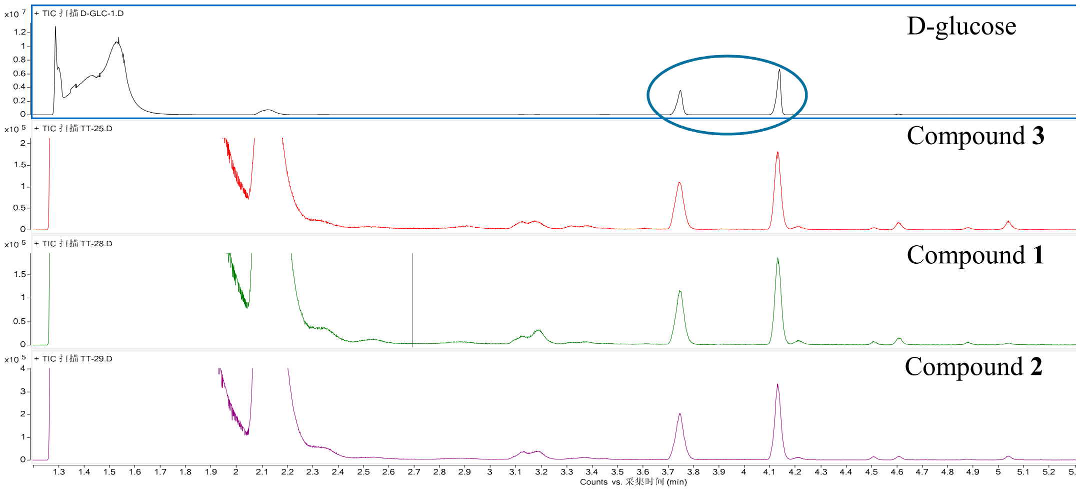

Fig. 25S Chromatography of D-glucose and the acid hydrolysis products of compounds **1-3** separated by GC-MS, the conditions of GC-MS: Agilent 5977A MSD; column, agilent DB-5ms (30m x 250μm, 350℃), program warming (180℃-250℃, 15℃/min).
